# Dual Electrochemical Treatments to Improve Properties of Ti6Al4V Alloy

**DOI:** 10.3390/ma13112479

**Published:** 2020-05-29

**Authors:** Stefano Rossi, Luciana Volgare, Carine Perrin-Pellegrino, Carine Chassigneux, Erick Dousset, Marielle Eyraud

**Affiliations:** 1 Department of Industrial Engineering, University of Trento, via Sommarive 9, 38123 Trento, Italy; luciana.volgare@alumni.unitn.it; 2 Matériaux Divisés, Revêtements, Électrocéramiques (MADIREL), Centre National de la Recherche Scientifique (CNRS), Aix Marseille Université, UMR 7246, 13013 Marseille, France; carine.chassigneux@univ-amu.fr (C.C.); marielle.eyraud@univ-amu.fr (M.E.); 3 l’Institut Matériaux Microélectronique Nanoscience de Provence (IM2NP), Centre National de la Recherche Scientifique (CNRS), Aix Marseille Université, UMR 7334, 13013 Marseille, France; carine.perrin@univ-amu.fr; 4 Institut des Sciences du Mouvement (ISM), Aix Marseille Université, UMR 7287, 13013 Marseille, France; erick.dousset@univ-amu.fr

**Keywords:** TiO_2_ nanotubes, bulk thermal treatment, bulk oxide layer, corrosion, wettability

## Abstract

Surface treatments are considered as a good alternative to increase biocompatibility and the lifetime of Ti-based alloys used for implants in the human body. The present research reports the comparison of bare and modified Ti6Al4V substrates on hydrophilicity and corrosion resistance properties in body fluid environment at 37 °C. Several surface treatments were conducted separately to obtain either a porous oxide layer using nanostructuration (N) in ethylene glycol containing fluoride solution, or bulk oxide thin films through heat treatment at 450 °C for 3 h (HT), or electrochemical oxidation at 1 V for 3 h (EO), as well as combined treatments (N-HT and N-EO). In-situ X-ray diffraction and ex-situ transmission electron microscopy have shown that heat treatment gave first rise to the formation of a 30 nm thick amorphous layer which crystallized in rutile around 620 °C. Electrochemical oxidations gave rise to a 10 nm thick amorphous film on the top of the surface (EO) or below the amorphous nanotube layer (N-EO). Dual treated samples presented similar results with a more stable behavior for N-EO. Finally, for both corrosion and hydrophilicity points of view, the new combined treatment to get a total amorphous N-EO sample seems to be the best and even better than the partially crystallized N-HT sample.

## 1. Introduction

Pure titanium is a polymorphic material and can crystallize into two types of crystalline structures: α and β [1].

The α-titanium shows a stable hexagonal close packed structure (hcp) up to about 880 °C while the β-titanium has a body-centered cubic structure (bcc) and is stable in the temperature range between 880 °C and the melting temperature of 1670 °C. Aluminum is the main stabilizing element of the alpha phase while vanadium is one of the most used isomorphic β-stabilizing elements thanks to its high solubility in titanium [1].

Ti6Al4V commercial alloy is biphasic with the following chemical composition: 6 wt% of aluminum (α-stabilizer) and 4 wt% of vanadium (β-stabilizer). In recent decades this alloy has been widely used in various fields and multiple applications thanks to its excellent mechanical properties, osseointegration properties, low density associated with high resistance to corrosion, wear, fatigue, creep, and to the propagation of cracks [2]. Considering medical applications, dental implants, as well as knee and hip prostheses are often made with this alloy. In particular, the biocompatibility is linked to the fast formation of an oxide surface layer of 2–5 nm [3] which is formed when the material is exposed to air or to any oxidizing medium such as human body fluid. This layer is protective because it slows down the corrosion process and is also able to reform if damaged in a few milliseconds [4]. It is composed by different oxides layers: a TiO layer directly in contact with the titanium alloy substrate; an intermediate Ti_2_O_3_ oxide and finally an outer TiO_2_ layer [5]. Unfortunately, the layer that naturally forms in contact with air is not completely stable and at a microscopic level, in particular media and conditions, it can be broken by triggering localized corrosion. Since patient lifetime increases, corrosion resistance of the prosthesis has to be increased to avoid a new surgery on older patients [6]. Moreover, as the material shows the tendency to form a passive layer which greatly reduces its reactivity, bonds between bone cells and prosthesis are not easily created, leading to adhesion loss with time [7]. In order to overcome these problems linked to bio-corrosive resistance and osseoinduction, surface modifications are necessary.

To increase the corrosion resistance properties, a dense oxide layer is generally grown on the metallic surface. It can be achieved by thermal oxidation [8,9], anodic oxidation [10,11], pulsed laser deposition [12], and reactive sputtering [13]. Among them, thermal and anodic oxidations can be considered as the simplest and cost effective techniques to generate a dense oxide barrier. Nanostructurated oxide layers could enhance the corrosion resistance of implants too. This effect is still not clear while a benefit [2] as the opposite result [14,15] is mentioned. Nevertheless, such surface treatment could enhance mechanical interlocking between prosthesis and bone. Barranco et al. [16] observed that osteoblasts showed a higher adhesion to surfaces by increasing their roughness. Several treatments can be realized such as plasma-spraying, grit-blasting with ceramic particles, sol-gel deposition, acid etching, or anodization in fluoride containing electrolyte [17,18,19,20,21,22,23,24]. This last method allows the formation of an amorphous and homogeneous TiO_2_ nanotube (TiO_2_ NT) array [25,26] with controllable dimensions depending on the experimental conditions. The well-known formation mechanism is a competition between the TiO_2_ growth thanks to anodization and its chemically oriented dissolution due to fluoride ions attracted by the positive anodic surface [27]. Different organic or inorganic electrolytes can be used: organic baths containing glycerol or ethylene glycol, and NH_4_F and H_2_O are often preferred to the aqueous ones that contains HF. This is mainly linked to the fact that they are less dangerous and lead to more homogeneous tubes although partially covered with a layer consisting of some conglomerates of partially dissolved tubes [28]. A systematic increase of the corrosion resistance was reported for a nanostructurated layer after heat treatment. However, an open question is related to the action mechanism of this thermal treatment. Some authors found the cause in the formation of a barrier layer that grows below the nanotubes [29,30,31,32]. On the contrary, Munirathinam et al. and co-authors concluded that the crystallization of the initially amorphous nanotubes was responsible for increasing the corrosion behavior [33].

To verify the effect of a barrier layer avoiding the crystallization of the nanotube, this paper proposes for the first time to electrochemically grow this barrier layer coupled to nanostructured film.

In addition, in most papers, only one kind of process is realized. When two processes are combined, few different experimental conditions are used to modify the layer. Moreover, the effect of each treatment is not studied separately. For example, in [2] where Grotberg et al. compare bare, nanostructured, heat treatment and nanostructured treatments combined with heat treatment, only one experimental condition is used. In [31,34] heat treatment of anodic NT was studied at different temperatures (but no variation for the NT layer is performed). 

In this research, a comparative study was made between the bare Ti6Al4V alloy (B) and the following surface treatments done to increase both osseointegration and corrosion resistance:

(1) Nanostructurated oxide layer (N), electrochemically obtained in ethylene glycol containing fluoride ions media;
(2) Bulk oxide, grown either by heat treatment (HT) in air at 450 °C or by electrochemical anodization (EO) in a sodium sulphate bath;
(3) Dual treatments combining nanostructuration followed by heat treatment (N-HT) or electrochemical oxidation (N-EO). To verify the effect of a barrier layer avoiding the crystallization of the nanotube that should arise in the case of N-HT, we propose for the first time nanostructuration followed by bulk electrochemical oxidation.

Therefore, our goal was to study the effect of each layer separately (HT, EO, N) or combined (N-HT, N-EO) and compare the morphological, structural, wettability, and corrosion results to those obtained from the bare surface (B). To determine the thermal stability and to characterize the phase transitions under heat treatment, non-isothermal methods (in-situ XRD) are also used in oxidizing media (air).

## 2. Materials and Methods 

### 2.1. Materials Synthesis and Post-Treatments

A biphasic α + β Ti6Al4V bar with a diameter of 1.4 mm (French company Aubert & Duval, Paris, France) was used as starting material. It was cut into disks with a thickness of about 2 mm, polished on SiC paper from a grade ranging from 180 to 4000, and then polished with 6 μm to 1 μm thick diamond pastes. An ultrasonic cleaning procedure was carried out in pure ethanol for 5 min. Finally, the bare samples were dried with compressed air. Bare titanium alloy samples were labeled as B.

TiO_2_ nanotubes were grown by electrochemical anodization using a two-electrodes configuration with a Ti alloy disk as working electrode placed in a PTFE holder (exposed area of 1.3 cm^2^) and a Pt grid as counter electrode. The optimum conditions in ethylene glycol solutions were selected according to literature [35]. Ethylene glycol electrolyte (VWR-chemicals, Fontenay sous Bois, France: GPR Rectapur) containing 0.3 wt% of NH_4_F (Sigma Aldrich 98%, Saint-Quentin-Fallavier, France) and 20 wt% of pure water was served as electrolyte at room temperature. A potential of 60 V was applied during 3 h using a power generator (Iso-Tech IPS 603, Paris, France). After anodization, the samples were rinsed with deionized water and dried. These nanostructured samples will be referred as N.

Compact layers were obtained either by heat treatment or bulk electrochemical oxidation. Ex-situ heat treatment under air was done on B and N samples at 450 °C for 3 h using a standard furnace (Nabertherm 30/3000 °C, Lilienthal, Germany). The heating rate was set to 5 °C/min. The heat treated samples were respectively referred as HT and N-HT. Electrochemical oxidation was performed in a 1 M sodium sulphate bath (Sigma-Aldrich Rectapur, Saint-Quentin-Fallavier, France). A classical three-electrode cell was used with a Pt foil as counter electrode, Ag/AgCl electrode as reference (0.2 V/NHE), and B or N samples as working electrodes. The samples were oxidized for 3 h at 1 V/(Ag/AgCl) and referred as EO and N-EO, respectively.

### 2.2. Characterization

Surface morphology and average composition were investigated using a scanning electron microscope (SEM) (Zeiss Gemini SEM 500 70-04, Oberkochen, Germany) equipped with X-ray energy dispersive spectrometer (EDS). Measurements were carried out using an acceleration voltage between 5 and 20 kV. To have a better understanding of the thickness of the layers, a thin cross section of the samples was realized using a dual beam Focused Ion Beam (FIB) (FEI- COMPANY/Helios 600 nanolab, Thermofisher Scientific, Hillsboro, OR, USA). Protective C and Pt layers were first deposited on the surface to protect it. A Ga+ ionic beam allowed to produce thin blades intended for microscopic examinations by SEM or transmission electron microscopy (TEM) (Tecnaï G2, Thermofisher Scientific) observations.

The structure of the various samples was analyzed using X-ray diffraction measurements. Two kinds of experiments were performed, both on a Philips X’Pert diffractometer (Malvern Panalytical, Malvern, UK) working in Bragg-Brentano geometry and using filtered CuKα (*λ* = 0.15418 nm) as a radiation source. Furthermore, θ–2θ scans were registered over a 2θ angular range from 20° to 80° with 2θ steps of 0.04° using a rapid detector, or from 24° to 59° for XRD in-situ experiments to save time. This 2θ range corresponds to a region where most of the diffraction peaks coming from titanium and titania (anatase, rutile) phases can be observed. Ex-situ acquisitions were performed at room temperature to investigate the changes in crystallinity before and after heat treatment. In-situ measurements were done to follow the structural changes occurring during the oxidization process. In order to dissociate the phase transition coming from the substrate from those coming from the nanotubes, experiments were performed on B and N samples. In-situ heating was done under air using a thermo-regulated furnace (HTK 1200 Anton Paar, les Ulis, France). A thermocouple was placed close to the sample surface and the error on the temperature determination was estimated to be around ±20 °C. A heating rate of 10 °C/min was applied from 25 °C to 280 °C, because no structure changes were expected in that range. Starting from 280 °C, the data were collected every 20 °C up to 760 °C. An equilibrium time of 1 min was accorded for temperature homogenization at each step. As the data collection time was equal to 5 min per pattern, this corresponded to an average heating rate of 2.5°/min. 

Anodic potentiodynamic polarization tests were carried out to determine the corrosion resistance properties of the initial B, N, HT, and EO samples as of combined treated samples: N-HT and N-EO. A three-electrodes cell was used in which the sample represented the working electrode, a Pt foil acted as the counter electrode, and an Ag/AgCl (KCl saturated) was used as the reference. All the potentials were indicated versus this reference. A Dulbecco’s Phosphate Buffered Saline (DPBS, phosphate buffered saline solution with KCl, KH_2_PO_4_, NaCl, Na_2_HPO_4_, MgCl_2_, CaCl_2_) solution (Sigma Aldrich) at 37 °C was used in order to simulate the human body environment. Polarizations were done after 30 min of sample immersion in test solution, at 2 mV/s from −0.1V/open circuit potential to 0.8 V/ref, as preconized in ASTM F2129—19a standard dedicated to determine the corrosion susceptibility of small implant devices. The tests were reproduced three times or more to assess the reproducibility. The mean value of the corrosion parameters obtained on three reproducible tests were evaluated and compared as a function of the surface treatments. 

Wettability tests were performed to determine the hydrophobic or hydrophilic superficial behavior of the surface in function of the conducted treatment [2]. The study of superficial energy deduced by the contact angle was connected to osseointegration, while a higher surface energy and therefore lower contact angle induced a better hydroxyapatite formation and an easier attachment of cells [32]. Wettability was determined by the measurement of contact angle between a 3-µL droplet of ultra-pure water and the surface of the samples. A contact angle video system consisting of special optics (Adimec MX 12P, Eindhoven, The Netherlands) and a camera was used. Data were analyzed using the Video Savant software 3.0 (IO Industries, London, ON, Canada). Contact angle measurements were recorded 5 s after the drop-off and repeated 10 times to assess the reproducibility and determine the error.

## 3. Results

### 3.1. Morphology 

**Figure 1:**
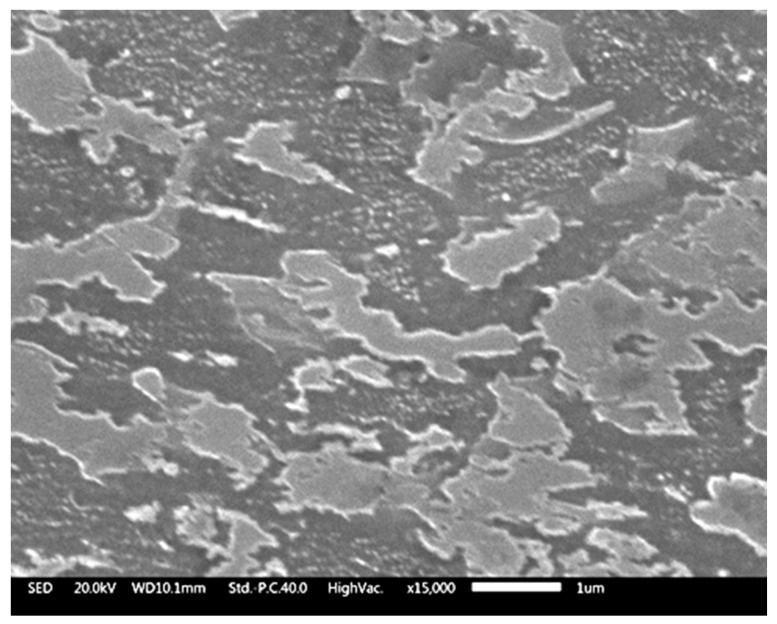
SEM SED image acquired on sample B after an etching in 4 mL HF + 2 mL HNO_3_ + 100 mL water.

Figure 1 is a SEM picture acquired on sample B’s surface after etching in 4 mL HF + 2 mL HNO_3_ + 100 mL water. Both α and β phases appeared, the whiter areas corresponding to the beta phase. EDS analysis (Table 1) made on white and dark zones confirms that result.

**Figure 2:**
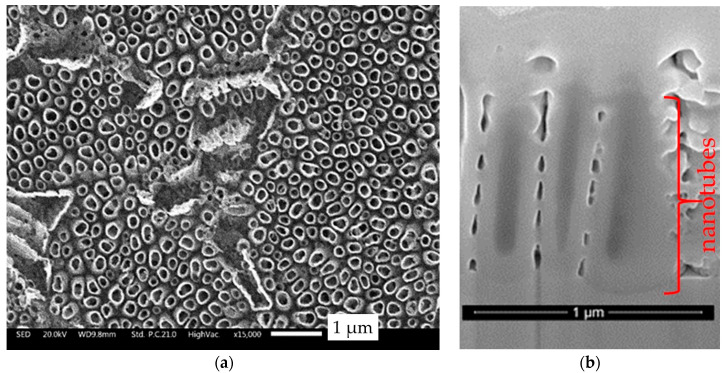
SED (secondary electron detector) SEM images of the TiO_2_ nanotubes (sample N) obtained on TiAl6V4 by electrochemical anodization in ethylene glycol containing fluoride solution. (**a**) Surface; (**b**) cross section.

The sample N surface appeared with a dull gray colored aspect. The morphology of the as-formed nanotubes is shown on the SEM images presented in Figure 2a,b. The surface is composed of a non-homogeneous array with a cylindrical geometry. The total diameter of the tubes is 275 ± 55 nm and their wall thickness is around 55 ± 5 nm. Some differences in height appeared with recessed areas. Only few references [36,37] dealing with this kind of inhomogeneity can be found. They suggest that the β phase is etched preferentially by fluoride ions due to a higher dissolution rate of the V-rich phase. The mean length estimated through cross section images (Figure 2b) is around 1 µm, varying from 0.8 to 1.2 µm, probably as a function of the phase from which they have grown. In order to have an idea of their stoichiometry, eliminating the substrate composition, the nanotubes were scratched on carbon tape. The EDS results given in Table 2 are in good agreement with TiO_2_ stoichiometry. Note that, as in the substrate, V and Al elements are still present in the nanotubes.

**Figure 3:**
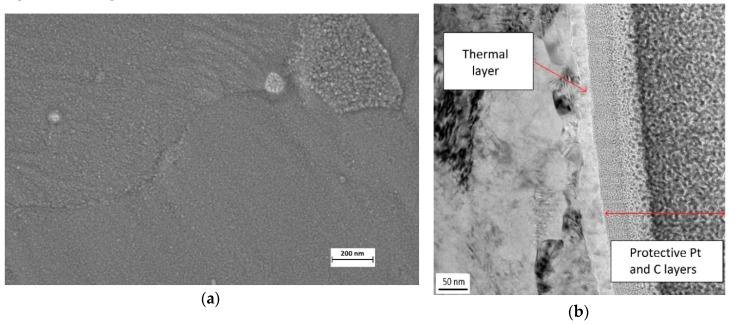
(**a**) SEM image of the heat treatment (HT) sample surface after 3 h at 450 °C; (**b**) cross section image obtained by TEM.

Bulk films made by heat treatment (sample HT) are composed by a very dense layer with a grain size between 5 and 10 nm (Figure 3a). A golden purple color depending of the incident light is clearly visible by eye on HT. In view of the literature, their thickness should be between 10 to 40 nm [4]. A mean value of 30 nm was actually estimated through the TEM image made on the cross section (Figure 3b) in agreement with [4]. 

**Figure 4:**
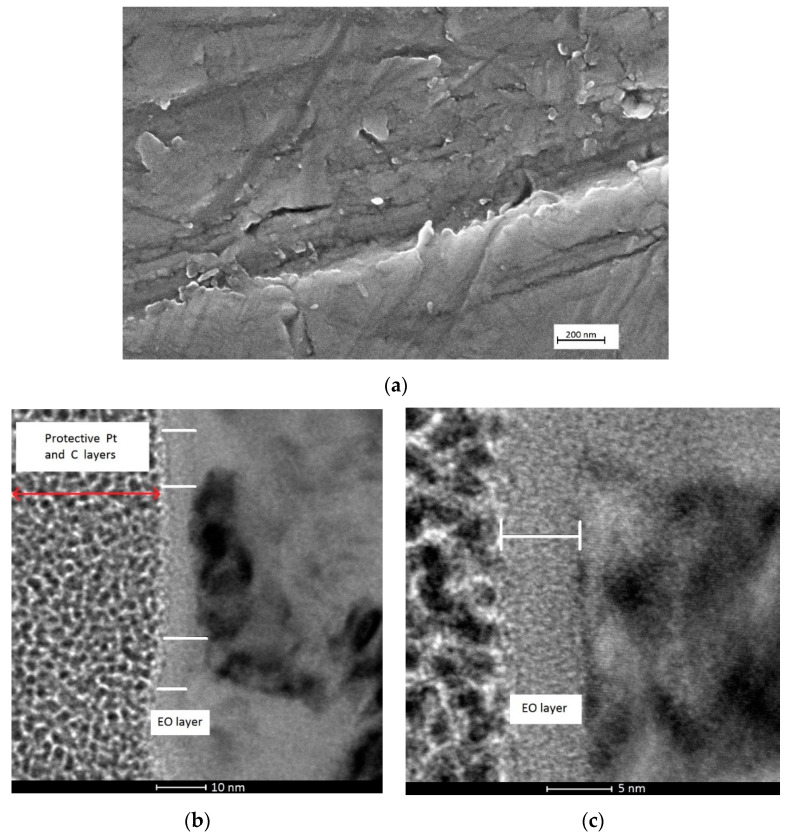
SEM image of the electrochemical oxidation (EO) sample surface (**a**); TEM cross section images on bulk electrochemical oxide EO sample obtained after 3 h at 1V/Ag/AgCl in sulphate solution: (**b**) low magnification; (**c**) high magnification.

In the case of bulk layer obtained by electrochemical oxidation, no change in color appeared for EO. SEM observations showed a smooth surface (Figure 4a). The grain size cannot be easily determined at this magnification that corresponds to that used for the HT sample (Figure 3a) for comparison. TEM observations on the cross section revealed a layer between 6 to 10 nm (Figure 4b), with an amorphous nature (Figure 4c) as confirmed later by XRD analysis.

**Figure 5:**
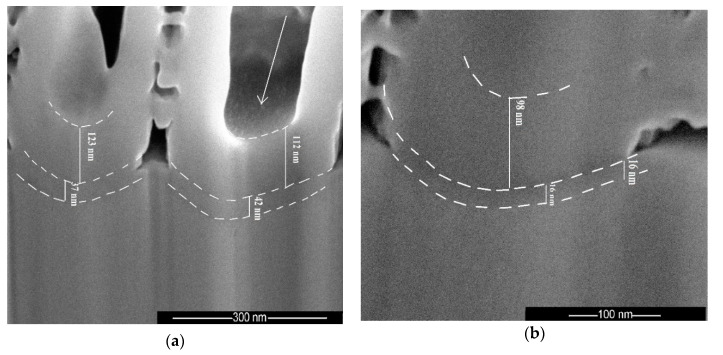
SEM cross section images of TiO_2_ nanotubes structures: (**a**) on nanostructuration (N)-HT after heat treatment at 450 °C during 3 h; (**b**) on N-EO after bulk electrochemical oxidation.

Dual treatment consisting of a porous layer followed by bulk oxide layer was realized to obtain N-HT and N-EO samples (Figure 5a,b, respectively). On the N-HT sample, no significant morphological changes were observed after heat treatment, with the nanotube array preserved. Nevertheless, on the SEM cross sectional view in Figure 5a, a start of crystallization seems to appear at the bottom of the tube due to the small crystallites (pointed at by the arrow). The tubes are now attached to the substrate by an almost 39 nm thick heat treatment layer underneath the tubes. In comparison with the literature, the thickness is lower because the temperature and time used for the heat treatment are lower too [38]. Velten et al. [4] shows that on titanium and its alloys the thickness of the heat treatment oxide layer increases in the same trend. A logarithmic function of up to 500 °C and parabolic for higher temperatures are individuated. For the same heat treatment conditions like those used in this study (450 °C, 3 h), but on pure titanium, the TiO_2_ thickness should be around 40 nm [4], which is in good accordance with our result.

On the N-EO sample (Figure 5b) the bulk oxide layer below the nanotubes is thinner (around 16 nm). The thickness determined for the bulk HT and EO layers on N samples are in good accordance with the values obtained from sample B.

### 3.2. Structural Analysis

**Figure 6:**
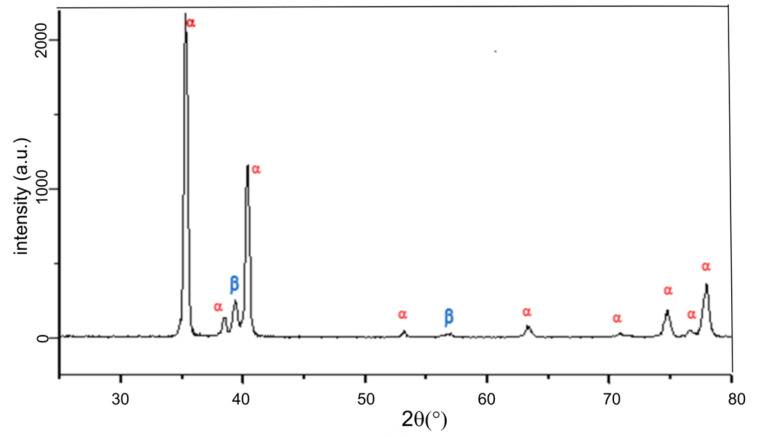
Ex-situ XRD patterns performed at room temperature (RT) on sample B. α and β phases coming from the corresponding JCPDF files of Ti α (PDF2 00-44-1294; ICDD, 2002) and Ti β (PDF2 01-089-4913; ICDD, 2002).

XRD pattern performed on sample B is presented in Figure 6. Only the diffraction lines of the α and β Ti substrate are evidenced (PDF2 44-1294; ICDD, 2002 and PDF2 01-089-4913; ICDD, 2002, respectively), with a small shift of the peaks towards higher 2*θ* values. This difference is attributed to the decrease in crystalline parameters of the alloy with respect to pure Ti due to a smaller atomic radius of V (0.135 nm) and Al (0.143 nm) against Ti (0.147 nm).

**Figure 7:**
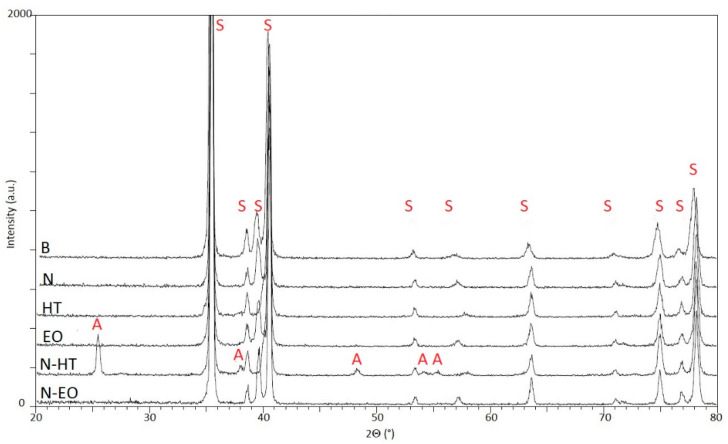
Ex-situ XRD patterns performed at room temperature (RT) on different samples. Bragg peaks of the various phases are indexed A for anatase and S for Ti substrate α or β phases.

The treated N, HT, EO, N-HT, and N-EO samples and B set as reference are presented in Figure 7. On sample N, the absence of a diffraction line coming from the TiO_2_ nanotubes structure is consistent with their reported amorphous nature [29]. 

On HT, EO, and N-EO no other diffraction lines than those coming from the substrate can be observed. Bulk TiO_2_ layers are still amorphous or the amount of crystalline phase is too low to be detected in our set-up. On the contrary, for N-HT, the heat treatment leads to the onset of a new phase since peaks are evidenced at 25.2°, 37.8°, 48.1°, 53.9°, and 55.1°. They correspond respectively to the 101, 004, 200, 105, and 211 peaks of the anatase phase (PDF2 021-1272; ICDD, 2002). From those results, it can be concluded that the initially amorphous TiO_2_ nanotubes have crystallized into anatase after a heat treatment at 450 °C. On the contrary, an anatase phase is not evidenced for HT. This is in accordance with the fact that anatase can only be stabilized in the nanosized range while rutile is the stable phase for bulk material [39,40].

Finally, among the samples used in this comparative study, only N-HT was partially crystallized, while at 450 °C the initial amorphous nanostructured layer moved in anatase and the thermal layer was still amorphous.

**Figure 8:**
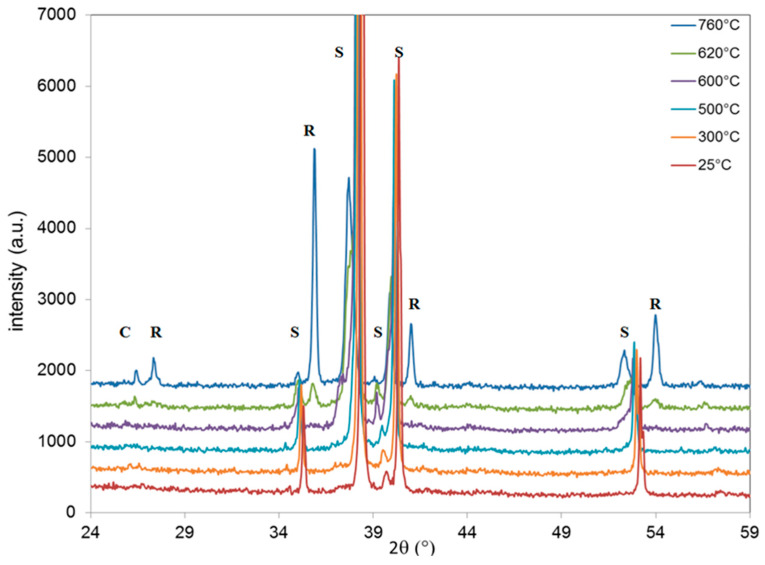
In-situ XRD pattern obtained for the B sample. The experiments were performed under air. Bragg peaks of the various phases are indexed R for rutile, S for Ti substrate, and C denotes the thermocouple sheath.

**Figure 9:**
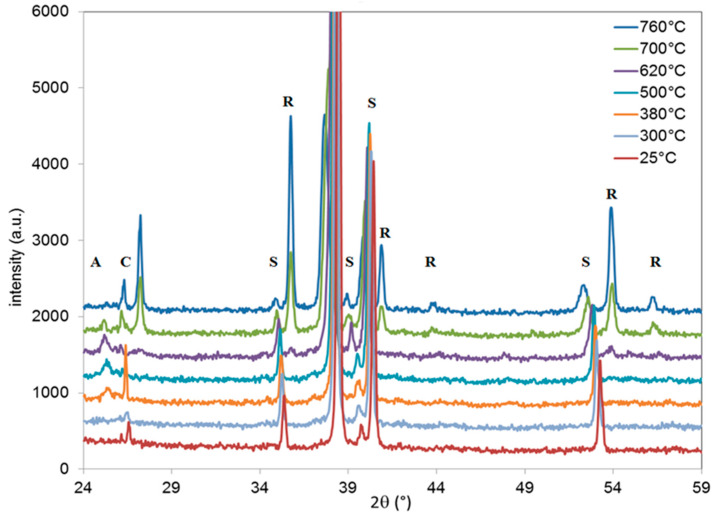
In-situ XRD pattern obtained for the N sample. The experiments were performed under air. Bragg peaks of the various phases are indexed A for anatase, R for rutile, S for Ti substrate, and C denotes the thermocouple sheath.

In order to get insight into the structural changes undergone during heat treatment in air, in-situ XRD experiments were performed on B and N samples (Figure 8 and Figure 9, respectively, where only some diagrams were gathered in the interest of the figure).

For sample B (Figure 8), at room temperature, only the diffraction lines coming from the Ti substrate are observed, as already mentioned. Note that the diffraction peak referred as “c” observed around 26.6° is related to the sheath of the thermocouple placed near the sample. The temperature increase leads to a shift toward lower diffraction angles of the diffraction lines coming from the Ti substrate. This can be explained by the thermal expansion of the Ti crystal lattice. At 620 °C, the onset of several diffraction peaks (noted as R) can be attributed to the rutile phase according to the JCPDS file (PDF2 021-1276; ICDD, 2002). The peak at 36.0°, attributed to the (101) plane, is the most intense peak due to the preferential orientation of the layer. A further temperature raise leads to an increase of the rutile signal. This suggests that the thickness of the Ti oxide barrier layer that has transformed into rutile continues to increase with temperature. 

For sample N (Figure 9), as already seen with ex-situ XRD experiments, no other peaks than those from the Ti substrate and the ceramic sheath of the thermocouple are observed up to 380 °C. At this temperature, a new diffraction peak appears at 25.2°, attributed to the (101) plane of anatase. At 620 °C, the diffraction peaks from the rutile phase are evidenced and their intensity increases with the temperature. At higher temperatures, the intensity of the peaks coming from R continues to increase while that for anatase decreases.

**Figure 10:**
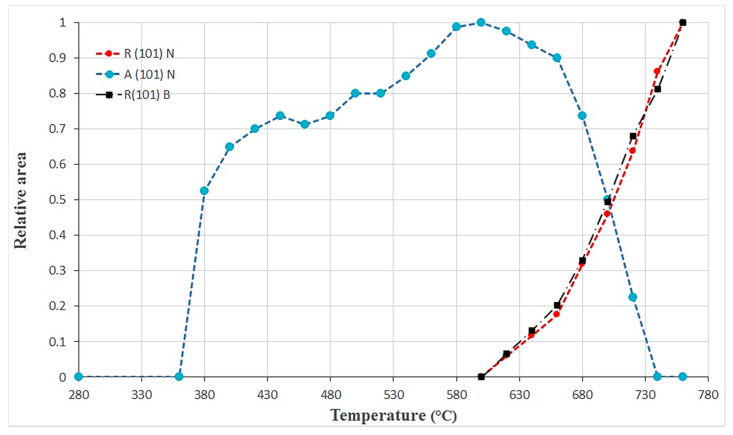
Estimation of the relative amount of the anatase and rutile phases deduced from in-situ XRD experiment performed under air on B and N samples. The estimation was done using the areas of the most intense Bragg peaks of each phase: 101 for anatase and 110 for rutile.

In order to quantify the relative proportion of the anatase and rutile phases, the areas of the most intense diffraction peak of each phase were calculated as a function of the temperature for both B and N samples. The results are summarized in Figure 10. The signal coming from rutile increases rapidly from 620 °C on both samples. The evolution of the anatase amount can be divided into three zones: a sharp increase from 300 °C to 440 °C, a slower evolution of up to 640 °C, and a decrease at higher temperatures. Anatase is totally transformed into rutile after 740 °C, while the signal coming from rutile continues to increase. This result proves that the rutile phase is coming mostly from the crystallization and growth of the thermal layer than from the anatase-to-rutile nanotube transformation. Note that the heat treatment temperature used in this study is 450 °C.

### 3.3. Corrosion Behavior

The corrosion study was done in a simulated human environment to evaluate the improvement of each surface treatment for the titanium alloy lifetime. 

**Figure 11:**
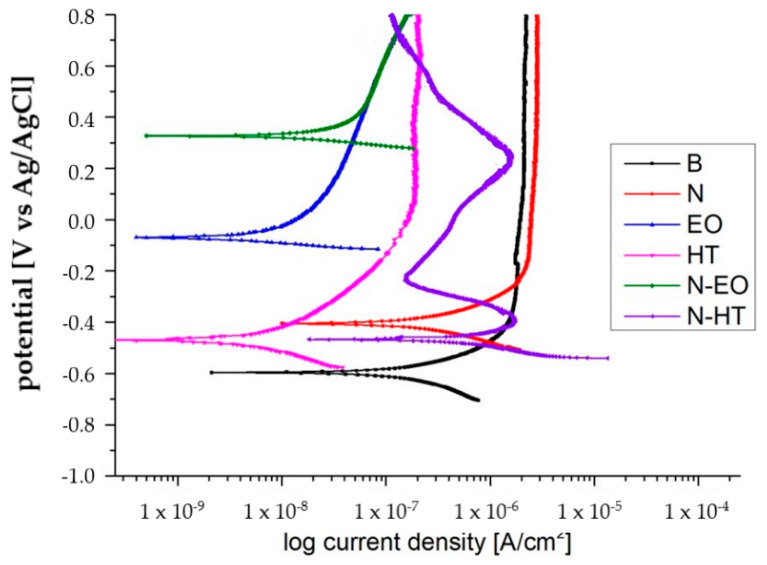
Anodic polarization curves obtained in a Dulbecco’s Phosphate Buffered Saline (DPBS) solution at 37 °C, on B, N, HT, N-HT, and N-EO.

The polarization curves were gathered in Figure 11 for sample B and surface treated samples. The results extracted from these curves are given in Table 3.

The corrosion potential (E_corr_) and the corrosion current density (i_corr_) were obtained using the Tafel’s slope method using the intersection of the cathodic slope with a line crossing E_corr_. Indeed, with the anodic part under passivation, the Tafel method is not valid. The passivation current density (i_p_) was determined at 0.8 V; the corrosion rates v_corr_ were deduced using the Faraday Law:

(1) $$V_{\mathrm{corr}}=\frac{M\cdot i_{\mathrm{corr}}}{\mathrm{nF}\rho }$$

with M as the Ti molar mass, i_corr_ the corrosion current density, F = 96,500 C mol^−1^, n the number of exchanged electrons, and ρ the density of Ti.

For any sample, the instantaneous corrosion currents were very low leading to very weak corrosion rates between 0.06 and 9 µm·y^−1^, with all Ti alloy surfaces in a passive state. The N-HT sample shows a higher corrosion rate in comparison, indicating a greater tendency to be corroded. However, it should be considered that the current remains at very low values and, considering the polarization curve form, the tendency to form a passive film with the decrease in current density is present. B and N samples were similar from a corrosion point of view, even if N is slightly worse (higher i_p_, higher i_corr_, and v_corr_), showing that the porous nanotube array could not improve the sample corrosion performance by itself. These results are in good accordance with those obtained in [14], pointing out that the nanotubes provide more channels for the electrolyte to reach the thin barrier layer at the bottom of the tubes. On the contrary, bulk layers made either by heat treatment or electrochemical treatments really improve the alloy corrosion resistance properties, increasing the corrosion potential and decreasing i_corr_, v_corr_, and i_p_ by a factor higher than 10. In the case of dual treatments, the bulk layer underneath the tubes shifts the corrosion potential into the noble direction. The oxidation treatment favors a passivation layer formation below the nanotubes that decreases i_p_ even if a non-stable behavior was obtained on N-HT. Possible defects could then be formed with the polarization with an increase of the current density. Nevertheless, at 0.8 V a high decrease in the passivation current density is still performed, finally indicating a more stable passive layer than those obtained on B and N samples. The improvement of the corrosion resistance is thus linked to the dense TiO_2_ layer due to heat treatment or electrochemical oxidations of the titanium substrate and not to the crystallization of the nanotube during heat treatment, as mentioned in [33].

### 3.4. Wettability 

**Figure 12:**
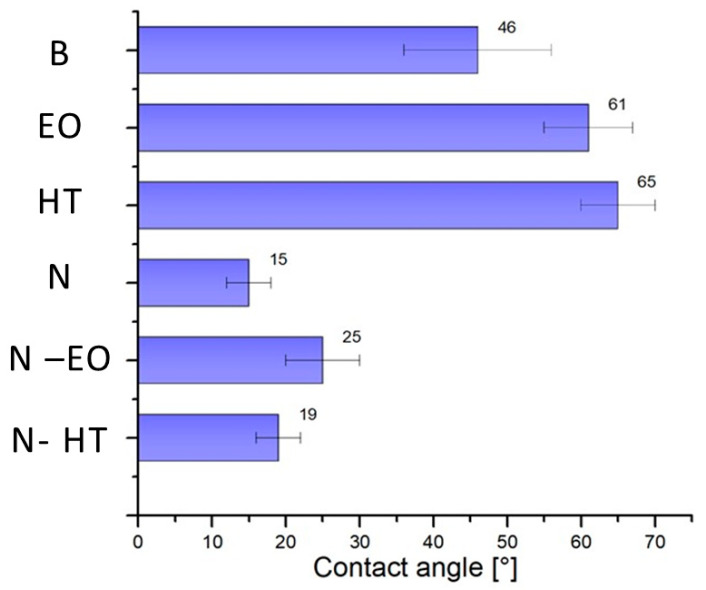
Contact angle determined on bare surface (B), EO, HT, N, N-HT, and N-EO.

The wettability is strongly connected with the surface roughness as indicated in the literature [41,42,43]. Surface wettability represents a key parameter: a high hydrophilicity, in fact, increases the surface energy and therefore the osseointegration. The hydrophilic properties quantified by water contact angle (WCA) is reported in Figure 12. The surface energy Es can be calculated according to Equation (2) [32,44]:

E_s_ = γ cos (θ) (2)

with γ = 72.8 mJ/m^2^ representing the surface energy between water and air at 20 °C, and θ representing the static contact angle accessible from those measurements. 

The non-treated sample presents a hydrophilic behavior with a contact angle value of 46 ± 10° and a surface energy of 50 ± 9 mJ/m^2^. The nanostructurated surface allowing water penetration into the tubes shows the lowest contact angle and therefore the highest surface energy. This increase in the hydrophilic characteristic of the samples can also be attributed to the increase of surface area available for adsorption due to nanostructuration but also to the nature of the interaction (amorphous surface vs. crystallized surface). Such an increase in hydrophilicity is very important for cell adhesion properties and is in good accordance with [32,45]. HT and EO with a compact layer show the lowest surface energy, while samples with dual treatments still conserve good hydrophilicity properties because of the porous layer at the surface.

## 4. Conclusions

Systematic morphological, chemical, structural, hydrophilicity, and electrochemical studies were conducted to determine the effects of nanostructuration N, heat treatment HT, bulk electrochemical oxidation EO, and combined treatments (N-HT and N-EO) on composition and functional properties of Ti-based alloy.

A nanostructured layer, grown by electrochemical oxidation in ethylene glycol containing fluoride bath, presented amorphous homogeneous titania nanotube array that crystallized into an anatase phase at 380 °C. At temperatures higher than 620 °C, a rutile phase was obtained too, mostly due to the thermal oxide crystallization underneath the nanotubes instead of the anatase-to-rutile nanotube transformation. HT made at 450 °C for 3 h and EO in neutral media at 1 V for 3 h, led both to compact amorphous oxide layers with a respective thickness of around 30 and 10 nm. In good agreement, on combined treated samples, the compact oxide layer below the nanotubes was thicker on N-HT than on N-EO.

In terms of osseointegration properties, contact angle measurements showed that all nanostructurated surfaces led to an increase in hydrophobicity.

In regards to the corrosion resistance performance, nanostructuration alone did not bring any improvement, because of the porosity induced by the array. We highlighted the beneficial effect coming from the compact amorphous TiO_2_ layers grown on HT, EO, and the dual treated samples, with the N-EO sample being slightly better than N-HT. It was then definitively demonstrated that the increase in corrosion resistance performed on N-HT after heat treatment was not due to the crystallization of the initially amorphous nanostructured layer but due to the growth of an amorphous thermal layer below the tube.

Finally, for the first time, the combined N-EO sample obtained by nanostructuration followed by bulk electrochemical oxidation appeared to be the best choice to improve both functional properties (hydrophilicity and corrosion), using only electrochemical techniques.

## Figures and Tables

**Table 1:** EDS analysis made on white and dark areas from Figure 1. Values accuracy ±0.5.

Kind of Surface	V (wt%)	Al (wt%)	Ti (wt%)
White zone	5	6	89
Dark zone	2	6	92

**Table 2:** EDS analysis in at% of nanotubes scratched on carbon tape. Values accuracy ±0.5.

Element	Ti	O	Al	V
at%	27	67	4	2

**Table 3:** Values of corrosion parameters extracted from polarization curves (Figure 11) obtained on the untreated and treated surfaces.

	E_corr_ (V/Ag/AgCl)	i_corr_ (µA/cm^2^)	V_corr_ (µm/y)	I_p_ (0.8 V/Ag/AgCl) µA/cm^2^
B	−0.61	0.2	1.7	2
N	−0.39	0.3	2.6	2.5
HT	−0.47	0.007	0.06	0.3
EO	−0.09	0.015	0.13	0.2
N-HT	−0.47	1	9	0.12
N-EO	0.32	0.1	0.9	0.15
